# Food Tray Sealing Fault Detection in Multi-Spectral Images Using Data Fusion and Deep Learning Techniques

**DOI:** 10.3390/jimaging7090186

**Published:** 2021-09-16

**Authors:** Mohamed Benouis, Leandro D. Medus, Mohamed Saban, Abdessattar Ghemougui, Alfredo Rosado-Muñoz

**Affiliations:** 1 Laboratory of Informatics and Its Applications of M’sila (LIAM), Department of Computer Science, University of M’Sila, BP 166 Ichbilia, Msila 28000, Algeria; abdessattar.ghemougui@univ-msila.dz; 2 Department of Electronic Engineering, ETSE, University Valencia, Av. Universitat, s/n-46100, Burjassot, 46100 Valencia, Spain; leandro.d.medus@uv.es (L.D.M.); mosa2@alumni.uv.es (M.S.); alfredo.rosado@uv.es (A.R.-M.)

**Keywords:** hyperspectral imaging, data fusion, deep learning, food packaging, fault detection

## Abstract

A correct food tray sealing is required to preserve food properties and safety for consumers. Traditional food packaging inspections are made by human operators to detect seal defects. Recent advances in the field of food inspection have been related to the use of hyperspectral imaging technology and automated vision-based inspection systems. A deep learning-based approach for food tray sealing fault detection using hyperspectral images is described. Several pixel-based image fusion methods are proposed to obtain 2D images from the 3D hyperspectral image datacube, which feeds the deep learning (DL) algorithms. Instead of considering all spectral bands in region of interest around a contaminated or faulty seal area, only relevant bands are selected using data fusion. These techniques greatly improve the computation time while maintaining a high classification ratio, showing that the fused image contains enough information for checking a food tray sealing state (faulty or normal), avoiding feeding a large image datacube to the DL algorithms. Additionally, the proposed DL algorithms do not require any prior handcraft approach, i.e., no manual tuning of the parameters in the algorithms are required since the training process adjusts the algorithm. The experimental results, validated using an industrial dataset for food trays, along with different deep learning methods, demonstrate the effectiveness of the proposed approach. In the studied dataset, an accuracy of 88.7%, 88.3%, 89.3%, and 90.1% was achieved for Deep Belief Network (DBN), Extreme Learning Machine (ELM), Stacked Auto Encoder (SAE), and Convolutional Neural Network (CNN), respectively.

## 1. Introduction

As reported in a packaged food report, global sales of packaged food amounted to approximately 2.47 trillion USD in 2016, with food sales forecast to reach approximately 2.64 trillion USD by 2019. However, according to many organizations, they estimated that one-third of all food produced in the world is lost or wasted [1].

To deal with this situation, each stage in the lifecycle of food products needs to ensure the safety of the food and consequently leverage the supply chain food, respecting the short shelf life of products and reducing the associated costs during the food lifecycle [2]. Thus, packaging technology is considered a vital step to ensure the quality of the food and prevent human poisoning. Barnes et al. [3] describe a new laser-based sealing system. They explored the integrity of semi-rigid sealed polymer food packages, and they assessed the performance of laser scatter imaging and polarized light stress images on the package food [4]. A database of 117 translucent tray seals was used, achieving 96% for laser scatter and 90% for polarized light in a database of 117 translucent tray seals. After that, different imaging techniques were investigated, including the active IR (infrared) thermography to inspect food tray sealing faults [5].

The rice samples quality is analyzed using Visible Near InfraRed (VNIR) hyperspectral imaging technology in [6]. Firstly, they cleaned the image samples from the scatter effect phenomena by carrying out five methods: SG1, SG2, MSC, SNV, and PMSC. In order to reduce the data space, they developed a regression technique called PLSR to select the optimal wavelengths among the full band wavelength to perfectly visualize the adulterated level of abnormal substances in rice samples.

The dianhong black tea was analyzed in [7] to check the quality. The image samples were collected by near-infrared imaging technology. The authors applied three methods: Iteratively Retaining Informative Variables (IRIV), Interval Random Frog (IRF), and Variable Combination Population (VCP) analysis to find the abundant wavelength. Feature extraction from images was obtained from the combination of two matrices and the proposed classifier achieved 99.6% accuracy.

Typically, the traditional imaging systems depict the object in the space color image (RGB space). Advanced imaging exploration have led to the expansion of the HyperSpectral Imaging (HSI) market, able to capture more properties (more spectral bands than a traditional RGB camera) from a single image. Indeed, food safety uses this technology to identify faulty products [5] in the manufacturing line.

For each image frame, data cubes generated by HSI sensors contain 3D data (W×H×D) corresponding to an image in size of *W* pixels in width, *H* pixels in height, and *D* spectral channels in depth, i.e., two dimensions in spatial information and one dimension in spectral information, usually called datacube. Then, 4D hyper cubes are generated if we consider time as the fourth dimension. The massive data collected in an HSI image (i.e., multi spectral band) requires a high computation for fault detection algorithms. However, food packaging is a fast process where many trays are processed per second, and then detection algorithms must be done in real time and detect tray quality on the fly while trays are circulating in the production line. To achieve a low computation time, machine learning and hand-crafted feature algorithms have successfully been applied on hyper-spectral images to keep the important features and ignore the redundant information [8].

To identify a small anomaly in a high dimensional image, a low-rank representation can be an effective tool to reduce the space and preserve the important information. For decades, Principal Component Analysis (PCA) was considered the best candidate among the machine learning algorithms to reduce the 2D data space [9]. However, its performance decreases when applied to 3D HSI datacubes as they converted into 2D space, which automatically leads to a loss of important information contained in the hyperspectral image. Sparse representation proposes an alternative for space reduction, which may handle the 3D datacube image while maintaining the relevant original space information [10], representing an image as a set of linear combinations of atoms from a dictionary that is made from training samples [11]. Chao [12] developed a hyperspectral–multispectral line-scan imaging system to distinguish wholesome and systemically diseased chickens. Successfully applied technologies for food quality and safety are described in [13]: hyperspectral imaging, magnetic resonance imaging, soft X-ray imaging, ultrasound imaging, thermal imaging, fluorescence imaging, and others. In their study, they argued that the cost of the equipment and lack of comprehension of imaging techniques suitable for food processing cannot meet modern manufacturing requirements. Meanwhile, they identified the principal key barriers in terms of software, hardware, and data availability (constraint budget, expensive and heavily imaging tools) that mostly limit the growth of the food industry. In [14], they investigated the potential of visible and near-infrared (VIS/NIR) HSI to distinguish between free-range and broiler chicken meats. They inspected 120 hyperspectral images of chicken fillets and extracted the spectral information from the Region Of Interest (ROI) and then applied Multiple Scatter Correction (MSC) to reduce the noise. They performed the Successive Projection Algorithm (SPA) to select optimal wavelengths from contiguous bands. To reduce the space, they applied the Principal Component Analysis (PCA) on the 3D datacube image. Then, a feature vector with 30 variables was extracted after applying the Gray-Level Gradient Co-occurrence Matrix (GLGCM). Finally, the Radial Basis Function-Support Vector Machine (RBF-SVM) achieved a Correct Classification Rate (CCR) of 93.33%. In [15], they used a multispectral imaging system to detect potato defects. They inspected seven types of potato defects collected through a multispectral camera with a spectral range of 676–952 nm. They applied preprocessing steps (simple threshold and morphological filtering), and only three wavelengths (690, 757, and 927 nm) were selected to make up a new image. Their model accuracy achieved 94.36% for defective potatoes. On the other hand, the Least Square-Support Vector Machine (LS-SVM) classification model using fused information, including spectral and texture features, achieved 90.70% for defective potatoes. In [16], they used the GLGCM method on 409×216×25 multispectral images of dried carrot slices with nine features introduced to the Support Vector Machine (SVM) classifier.

Remote sensing technology use the shallow and deep learning to explore the important patterns located in an hyperspectral image. However, non-contact inspection of heat seals in food trays using HSI and deep learning has been rarely studied so far. Handcrafted feature tools are made from a set of image processing methods to find both explicitly and implicitly the amount of spatial and frequency information located in low- and high-space information. For instance, it may learn multiple types of information from image or signal information including frequency component, color and texture, shapes, etc. Typically, these features are suitable to power the machine learning to accomplish the machine vision tasks (i.e., detection, classification, regression, segmentation) [9]. The advances in computer vision, including hyperspectral imaging in particular, often require cleaned hand-crafted feature extraction to accomplish the task well. In this context, the information is gathered from multi wavelengths, and consequently, a deep analysis is required to locate the relevant features either in spatial or frequency space. In [17], they demonstrated that deep learning can play a new role in the food science. Unlike the data processed with feed-forward neural networks by no more three layers, deep learning can analyze data (image, signal, video) through different layers, which can process them without any pre-processing step or having high corrupting noise levels. In the spectral imaging field, CNN showed more advantages than other deep learning techniques in hyperspectral image classification [18,19,20,21]. For instance, in [22], they studied the varieties of maize seed scanned by near infrared (NIR/visible) imaging technology. In the pre-processing stage, the image samples were segmented into small regions, called regions of interest (ROI). To distinguish the categories of maize, the CNN architecture was trained to account for the number of pixels located in the ROI.

Additionally, Principal Component Analysis (PCA) and the Auto Encoder (AE) classifier are combined for feature extraction and dimensionality reduction, achieving a good accuracy in HSI classification [23] with a reduced computational load. On the other hand, Long Short-Term Memory (LSTM), Deep Belief Networks (DBN), or adversarial networks have shown a great success in image classification, but few studies have investigated its application to hyperspectral images. The main drawback of deep learning methods lies in the hyperparameter fine-tuning optimization, requiring more data for the training process. In fact, many techniques have been used in building optimal network structures to fit the desired application [17].

In parallel with the aforementioned methods, there exist hyperspectral fusion techniques to reduce the dimensionality space, but in an ad hoc form [24], i.e., manually tuning the data fusion and feature extraction process. To the best of our knowledge, we have found a few applications for food inspection based on hyperspectral images because they either use a proprietary database that is not publicly available or they study commercial systems [25]. Then, existing works do not provide comprehensive comparisons with state-of-the-art food inspection techniques specifically targeted for food packing seals. In [26], they controlled the wheat image scanned by ultraviolet (UV) and White Light (WL) imaging techniques. First, for each imaging technique, Local Binary Patterns (LBP) and Gray-Level Co-occurrence Matrix (GLCM) methods process wheat grain image samples to extract a unique vector of size 13. Afterwards, various machine learning methods are applied to distinguish the wheat types (durum and bread). Second, they applied the Discrete Wavelet Transform (DWT) technique to fuse the images obtained by ultraviolet (UV) and White Light (WL). After that, Local Binary Patterns (LBP) and Gray-Level Co-occurrence Matrix (GLCM) methods are applied on the fused image to extract the important features. In the classification stage, the SVM and Multilayer Perceptron (MLP) methods were trained to extract the features that classify the wheat sample categories (durum and bread).

In our case, our first contribution is a novel hyperspectral food inspection algorithm based on a PCANet network [27]. The proposed machine learning algorithm detects any anomaly located in the seal by analyzing the mean value of spectral bands in the datacube. Three fusion rules are performed, averaging data over the hyperspectral image, giving robustness to anomaly variations due to the sequential capture of the hyperspectral bands or product contamination that may have been generated during the food production cycle.

In order to produce a quality assurance system and make the system more autonomous, this paper investigates the use of different fusions and deep learning techniques. The first technique is using the sensor fusion approach as the loss of spatial and spectral information can be overcome and the quality of the sealing process can be assessed. The result of the fusion algorithm is used as input to the machine learning model. Thus, the deep learning algorithm deals with low-dimensional data, accelerating the computation without reducing the detection ratio.

Existing band fusion algorithms operate by transforming hyperspectral data (3D) into a lower dimension (2D), which is similar to an averaging approach along the image bands [28]. However, the averaging approach does not take the spatial information into account and does not perform averaging along the spatial dimensions. In general, pixel relationship information is more discriminant and the frequency domain has the advantage of efficiently removing high component noise. Moreover, existing fusion techniques cannot effectively ignore the redundant information located among the existing bands [29,30]. This work analyzes those data fusion techniques suitable for the HSI and food tray characteristics.

Seal integrity, polarized light and laser scatter take full advantage of detecting faults in heat seals of food and have been widely used in the packaging industry [31]. However, due to the noise sensor and low intensity distribution, the detection performance degrades. An alternative technique to reduce the dimensionality is the spectral band selection. However, improper selection of bands may bring loss of information as well as false alarm detection accuracy. In order to overcome these drawbacks, the band image selection has to be studied. In this work, we avoid this step as it must be done manually and it is not the main focus of this work. We test different fusion strategies on the hyperspectral image datacube to create a single 2D pseudo-image, which is entered into the machine learning algorithm. Pixel-level fusion methods, including spatial and spectral information, are studied as they are computationally efficient and easy to implement.

In this study, two pixel-level fusion methods were employed: spatial image fusion and transform image fusion. The first method fused different channels into one single channel through spatial computation rules, and the second takes into account the spectral information by transforming the HSI image into a single image through frequency transformation tools. Both methods reduce the input data space and only keep important information. The fused data are then used as input for the deep learning (DL) classifier. Four different classifiers are analyzed in this study.

Using fused data reduces the computational cost of the algorithm while keeping a high classification accuracy.

## 2. Materials and Methods

**Figure 1:**
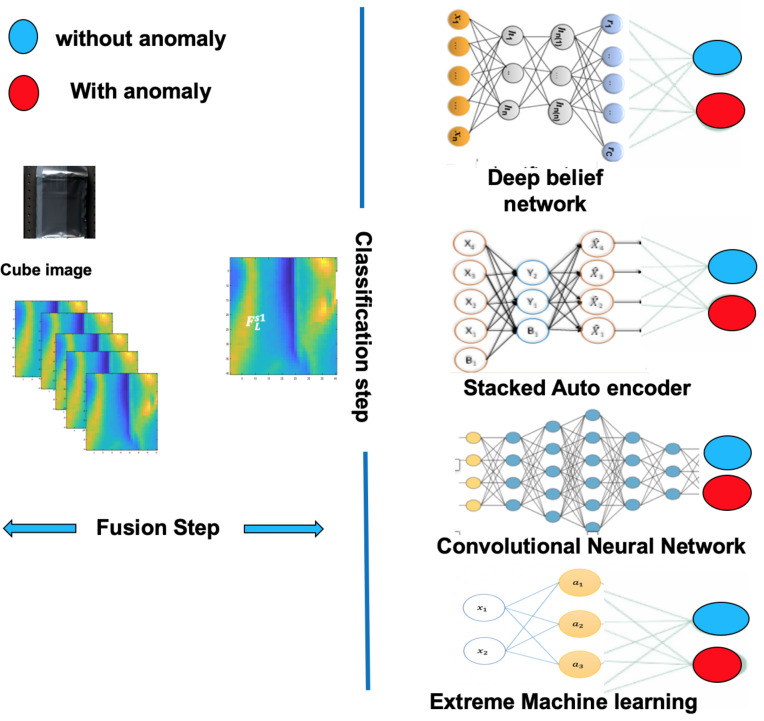
General framework of the food tray contamination detection based deep learning methods: first, data fusion on hyperspectral data; second, deep learning algorithm classification.

Figure 1 shows the block diagram of the proposed method. First, data fusion is applied to the 3D HSI image to obtain reduced data, which are used to feed a DL classifier. By doing this, the DL algorithm is smaller when compared to a DL algorithm with the full 3D HSI image as input. This fact makes it computationally efficient while maintaining high accuracy.

### 2.1. Dataset

A line scan camera composed of 410 lines by 320 pixels with 168 channels (wavelengths in the range from 891.1 to 1728.4 nm) was used to scan the food packaging. The hyperspectral camera of the HSI system used to acquire the images was a Pika NIR-320 (Resonon Inc., Bozeman, MT, USA). The vendor’s software was “Spectronon” Version 2.116, required to configure the camera. The image dataset was created by adding impurities to the package seal as they usually arise in the production line. Eleven impurities were created: washers, sugar, flange, plywood, cork, elastic rubber, wood, paper, silver paper, hair, and polarized plastic. Each data point was coded in a 16-bit signed integer. Thus, each tray hypercube image was 4.204 MiB in size.

Manual processing was made on the dataset by applying ROI filtering around a contamination area (more details can be found in the open source repository: https://github.com/LeoSf/HypLabTool (accessed on 1 September 2021)). As a result, a resized image of 40×40×168 was obtained. After evaluating the spectral information of tray samples, it was observed that the spectral ranges from 891.1 (nm) to 984.0 (nm) and 1631.2 (nm) to 1728.4 (nm) did not provide meaningful information. As result, 38 channels were removed and the obtained datacube contained 130-channels, from 984.2 to 1631.0 (nm).

**Figure 2:**
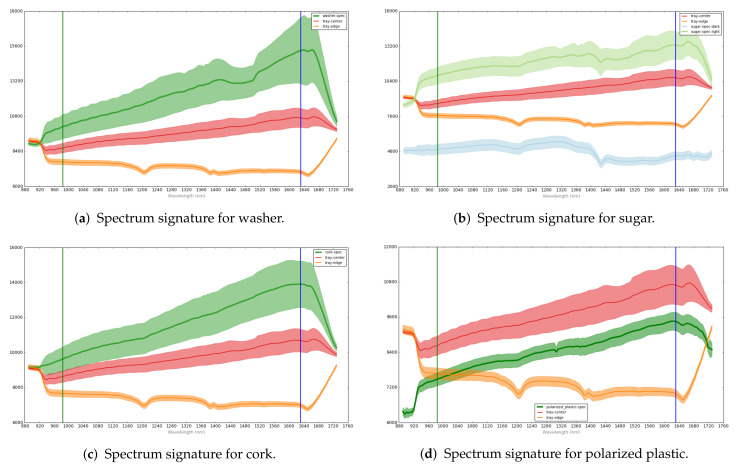
Spectrum signatures for some defects in food tray packaging in different tray areas: anomaly region (green), the edge region (tray—edge label, orange area), inner region of the tray (tray—center label, red area).

For each food tray, multiple non-overlapping Regions Of Interest (ROI) were extracted. Thus, a dataset was obtained, with 2112 valid ROIs from which 1123 were labeled as “normal” and 989 as “faulty”. Figure 2 shows the spectral signatures of some of the anomalies: washer, sugar, cork, and polarized plastic. For each of them, the curves represent the anomaly region (green area), the edge region without anomaly (tray-edge label, orange area), and an inner region of the tray (tray-center label, red area); the normal edge region and the tray center region are included as a comparison with the faulty region. Each curve corresponds to the mean values of the ROIs in the same class and its associated shadowed area in the same color corresponds to the standard deviation. As seen in the figure, the morphology of the curves depend on the class (anomaly, edge, or inner part). A total of 337 ROIs were kept for final validation, using 1775 ROIs for training.

### 2.2. Data Fusion Techniques

**Figure 3:**
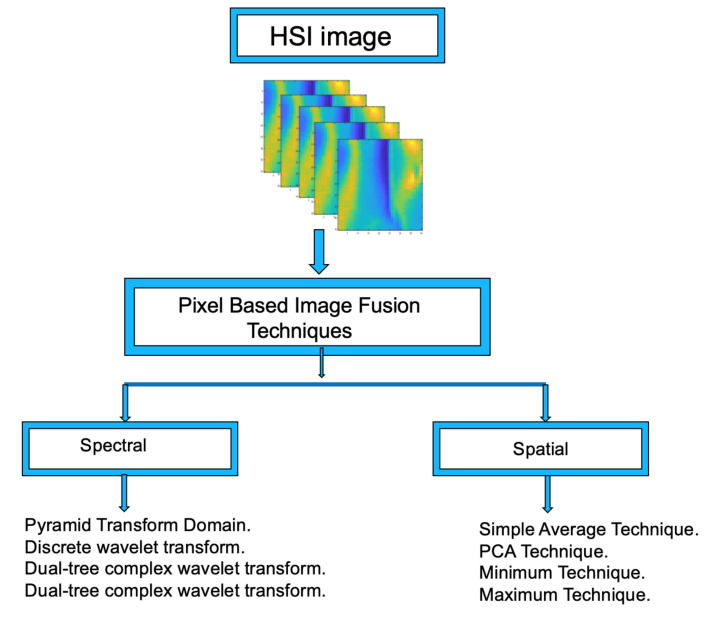
Spectral and spatial fusion methods used to reduce the number of hyperspectral data obtained from the HSI camera.

In order to reduce the extra computation burden and the “Hughes phenomenon” typically appearing during DL training, the dimension of the hyper-spectral images was transformed using different fusion methods. Figure 3 shows the data fusion procedures proposed in this work.

Spatial data fusion deals with pixel values of the 3D datacube HSI in which the pixels’ values are analyzed through a set of operators to produce a 2D data matrix equivalent to an image. The proposed procedures are: Minimum, Maximum, Simple Average, and PCA, which are described in detail below.

Let I(j,j,L) be a datacube containing the hyperspectral image, where *M* is the number of rows, *N* the number of columns, and *L* is the number of spectral bands, i.e., an M×N pixel array with L spectral channels for each pixel. The proposed data fusion procedures convert this datacube into a 2D data matrix F(i,j), which can be associated with an image, i.e., the hyperspectral image (3D) is transformed in a 2D data matrix (image).

#### 2.2.1. Spatial Data Fusion: Simple Average

Here, the fused image is computed by averaging pixels along the whole image band as described in Equation (1).

(1) $$\begin{array}{c} F\left(i,j\right)=I(i,j,\mathrm{Average}\left(L_{k}\right))),\;i=1..M,j=1..N,k=1..L \end{array}$$

#### 2.2.2. Spatial Data Fusion: Minimum Data Value

The lowest values in the spectral dimension *L* are selected for each of the pixels in the M×N array. Thus, a single image F(M×N) is generated, as shown in Equation (2).

(2) $$\begin{array}{c} F(j,j)=I(i,j,\mathrm{Min}(L)),\;i=1..M,j=1..N,k=1..L \end{array}$$

#### 2.2.3. Spatial Data Fusion: Maximum Data Value

The highest values in the spectral dimension *L* are selected for each of the pixels in the M×N array. Thus, a single image F(M×N) is generated, as shown in Equation (3).

(3) $$\begin{array}{c} F(i,j)=I(i,j,\mathrm{Max}(L)),\;i=1..M,j=1..N,k=1..L \end{array}$$

#### 2.2.4. Spatial Data Fusion: PCA Technique

This technique has been shown to be successful in space reduction and feature extraction tasks [32]. In this work, we have applied it on the HSI datacube image to produce a single image that preserves the maximum amount of feature details from each image band. The steps of the PCA data fusion technique are the following:

Step 1. A transformed matrix size A(i×j,L) is created from the HSI datacube.

Step 2. The covariance matrix is computed as given in Equation (4).

(4) $$\begin{array}{c} C=\mathrm{cov}\left(F\left(M\times N,L\right),F{\left(M\times N,L\right)}^{*}\right) \end{array}$$

Step 3. The eigenvalues and eigenvectors from the covariance matrix *C* are calculated.

Step 4. The eigen matrices P(L,d) are computed as the selection of the highest values of eigen values *d* and (d≤L).

Step 5. A 2D image is built by projecting the original matrix into the eigenvectors, computed as in Equation (5).

(5) $$\begin{array}{c} H(i,j,d)=A(i\times j,L)\times P(L,d) \end{array}$$

Step 6. Average all image matrices as given in Equation (6).

(6) $$\begin{array}{c} F(i,j)=H(i,j,\mathrm{Average}(d)) \end{array}$$

In spectral data fusion methods, each single image from the HSI image is decomposed into an effective coefficient along high and low space frequencies. In both cases, one single image obtained may handle discriminant features that will be used to further leverage the classification step. Specifically, given an M-dimensional hyper-spectral datapoint *I* , with *L* being the spectral dimension, $I=\{I_{1},\ldots ,I_{L}\}$, in which *L* is the number of bands located in the original data. Spectral fusion methods aim at transforming these images from the original space domain by firstly applying a transform function, then applying a data fusion function, and finally constructing the fused image, as described in Equation (7), where ψ and $\psi^{-1}$ are the transfer functions and its inverse.

(7) $$\begin{array}{c} F\left(i,j\right)=\psi^{-1}\left(F\left(\psi \left(I_{M\times N\times 1}\right),\psi \left(I_{M\times N\times 2}\right)\ldots \ldots \ldots \psi \left(I_{M\times N\times L}\right)\right)\right. \end{array}$$

In this paper, we adopted four spectral fusion techniques as explained below.

#### 2.2.5. Spectral Data Fusion: Discrete Wavelet Transform—DWT

This technique is employed to decompose the image or signal through a specific function, called the wavelet function, into a series of coefficients that yield high- and low-frequency information [33]. In the case of a signal, it is defined as in Equations (8) and (9), where $j>j_{0}$.

(8) $$\begin{array}{c} W_{\varphi }\left(J_{0},K\right)=\frac{1}{\sqrt{M}}\sum_{X}f\left(x\right)\varphi_{j_{0},k}\left(x\right) \end{array}$$

(9) $$\begin{array}{c} W_{\psi }\left(J_{0},K\right)=\frac{1}{\sqrt{M}}\sum_{X}f\left(x\right)\psi_{j,k}\left(x\right) \end{array}$$

The Inverse DWT (IDWT) is defined as in Equation (10), where $f\left(x\right),\varphi_{j_{0},k}\left(x\right)$ and $\psi_{j,k}\left(x\right)$, x=0,1,2,…,M−1.

(10) $$\begin{array}{c} \begin{array}{cc} f(x)= & \frac{1}{\sqrt{M}}\sum_{k}W_{\varphi }\left(j_{0},k\right)\varphi_{j_{0},k}\left(x\right) \end{array}\;\begin{array}{c} +\frac{1}{\sqrt{M}}\sum_{j=j_{0}}^{\infty }\sum_{k}W_{\psi }\left(\;j,k\right)\psi_{j,k}\left(x\right). \end{array} \end{array}$$

In case of an image, the approximation coefficients are located in four subbands: LL refers to the average information, and LH, HL and HH are considered as the image details. In this work, we have employed the DWT on the HSI datacube, which consists of N images. Such coefficients are derived from each band image and then can be combined through different rules to produce one reconstructed image (i.e., performing the inverse discrete wavelet transform (IDWT). In addition, the extracted coefficients can be merged through series of techniques rules [34]. In this work, we employed a technique called maximum value scheme.

#### 2.2.6. Spectral Data Fusion: Shift-Invariant Discrete Wavelet Transform—SIDWT

To tackle the shift variant issue of classical DWT, SIDWT was employed as a shifted wavelets channel to decompose the signal within tree decomposition. It gives a brief summary of SIDWT on the signal, which can be automatically expanded to the image representation. It is given as in Equations (11) and (12), where $w_{n+1}\left(n\right)$ and $s_{i+1}\left(n\right)$ are the wavelet and input sequence.

(11) $$\begin{array}{c} w_{n+1}\left(n\right)=\sum_{k}g\left(2^{i}\times k\right)\times s_{i}\left(n-k\right) \end{array}$$

(12) $$\begin{array}{c} s_{i+1}\left(n\right)=\sum_{k}h\left(2^{i}\times k\right)\times s_{i}\left(n-k\right) \end{array}$$

The approximation signal is composed by performing shift-invariant wavelet and scale sequence through appropriate filters $\tilde{h}\left(2^{i}\times k\right)$ and $\tilde{g}\left(2^{i}\times nk\right)$. It is given in Equation (13).

(13) $$\begin{array}{c} s_{i}\left(n\right)=\sum_{k}\tilde{h}\left(2^{i}\times n-k\right)\times s_{i+1}\left(n\right)+\sum_{k}\tilde{g}\left(2^{i}\times n-k\right)\times w_{i+1}\left(n\right) \end{array}$$

#### 2.2.7. Spectral Data Fusion: Dual-Tree Complex Wavelet Transform DT-CWT

The DT-CWT was inspired by Gabor Wavelets Transform processing scheme [35]. The DT-CWT employs two trees of real filters, tree “a” and tree “b”, which refer to the real and imaginary parts of the complex coefficients. Unlike Gabor wavelet transform, the DT-CWT filters are scaled to produce a high reconstruction. However, their directions are fixed. The DT-CWT expansion of an image $M(\vec{x})$ is given by Equation (14), where $i=\mp 15^{\circ },\mp 45^{\circ }$ and $\mp 75^{\circ }$. The scaling function $\varphi_{j_{0},k}$ and the wavelet function $\psi_{j,k}^{i}$ are complex. $w_{\varphi }\left(j,k\right)$ indicates the scaling coefficients and $w_{\psi }\left(j,k\right)$ are wavelet coefficients of the transform.

(14) $$\begin{array}{c} M\left(\vec{x}\right)=\sum_{k}w_{\varphi }\left(j_{0},k\right)\varphi_{j_{0},k}\left(\vec{x}\right)+\sum_{i}\sum_{j>j_{0}}\sum_{k}w_{\psi }\left(j,k\right)\psi_{j,k}^{i}\left(\vec{x}\right) \end{array}$$

Wavelet decomposition families are able to offer approximation of an image in low-frequency sub-bands that contain the major information from each image band. The main steps of wavelet fusion scheme are provided below:

Step 1: Suppose we have L images from the HSI datacube image.

Step 2: Perform three-level wavelet decomposition transform (i.e., DWT, SIDWT, and DT-CWT) on each image from the 3D cube image to obtain both low-frequency and high-frequency sub-bands at each level.

Step 3: At the three-level stage, perform the maximum value scheme value to obtain composite low- and high-frequency sub-bands of each image.

Step 4: Perform three levels inverse wavelet transform (i.e., DT-CWT, DWT, SIDWT) on the composite low- and high-frequency sub-bands to obtain the fused image.

#### 2.2.8. Spectral data fusion: Laplacian Pyramid—LP

The Laplacian Pyramid (LP) is considered an improvement of the Gaussian Pyramid (GP) [36]. GP transforms an image by applying the convolution and down-sampling operations. However, GP ignores the high-component-frequency Laplacian [37]. To deal with this issue, LP was proposed to preserve this information. In this paper, we have applied LP on the HSI image to decompose each image band into a series of coefficients that contain the important information and then fused them to produce it as single image. The computation process is explained below.

The input image is the bottom level of the GP and is called the first level, which is denoted as $G_{1}$. The *n*-th level image is denoted $G_{T}$, which is obtained by performing the Gaussian kernel convolution and down-sampling operation on the (T−1)-th level image, $G_{(}T-1)$. The process can be expressed in the Equations (15) and (16), where conv and $GP_{↓}$ indicate the Gaussian kernel convolution operation and down-sampling operation, respectively. The down-sampling operation resizes the image size to half the size of its lower level.

(15) $$\begin{array}{c} G_{1}=I\left(i,j,L=1\right) \end{array}$$

(16) $$\begin{array}{c} G_{T}=GP_{↓}\left(\mathrm{conv}\left(G_{T-1}\right)\right. \end{array}$$

#### 2.2.9. Spectral Data Fusion: Laplacian Pyramid Generation Using Gaussian pyramid

Each layer of LP is obtained by performing a subtraction as given as in Equation (17).

(17) $$\begin{array}{c} LP_{T}=G_{T}-\mathrm{con}v\left(GP_{↑}\left(G_{T+1}\right)\right. \end{array}$$

At each level, the size of image is scaled twice from the size of its upper level through an up-sampling operation, where $GP_{↑}$ denotes the up-sampling operation.

In this paper, the operation of LP transform is expressed by the following formulation as in Equation (18), where k=1,2,…,T and k=1,2,…,T.

(18) $$\begin{array}{c} I_{out}=LP\left(I\left(i,j,L=1\right),K\right) \end{array}$$

The image reconstruction is obtained through a series of coefficients from LP pyramid $\left[k_{1},\ldots ,k_{T}\right]$ by using the backward recurrence as expressed in Equation (19), where the up-sampling operation expands the image size from M×N to 2M×2N.

(19) $$\begin{array}{c} LP_{T}=G_{T}-\mathrm{conv}\left(GP_{↑}\left(G_{T+1}\right)\right. \end{array}$$

The fused image can then be produced through the value of maximum value from the LP. Thus, it is selected at each LP level from each image band as in Equation (20).

(20) $$\begin{array}{c} LP_{T}^{\max}=\mathrm{Max}\left(LP_{T},N\right) \end{array}$$

Finally, the fused image is obtained as in Equation (21).

(21) $$\begin{array}{c} F\left(i,j\right)=LP_{T}^{\max} \end{array}$$

### 2.3. Deep Learning Methods for Food Tray Classification Using Fused Data from Hyperspectral Images

In this section, we provide an overview of the deep learning architectures providing the best results in our specific detection problem: Convolutional Neural Network (CNN), Deep Belief Network (DBN), Extreme Leaning Machine (ELM), and Stacked Auto-Encoders (SAE).

Extensive research work has demonstrated the effectiveness of CNN in achieving a high accuracy without previous feature extraction steps. The CNN makes the image abstraction simpler than traditional computer vision methods. Thus, extended CNN architectures are proposed, including AlexNet, ZFNet, VGGNet, ResNet, GoogLenet, MobileNet, and DenseNet [38]. CNNs are made to overcome the data collection issue, reducing the computation training time, as well as obtaining meaningful features. In order to use 2D CNN architectures, the HSI data cube must be fused first to obtain a 2D representation. Then, the CNN architecture must be tuned with specific parameters (e.g., kernel size, padding size, and pool layer). The backpropagation algorithm and fine-tune procedure is performed to train the CNN. In our case, we have trained the dataset in a five-layer CNN, namely C1, C2, C3, C4, and C5. Layers F1 and F2 are the fully connected layers, and SoftMax is the classification layer. The complete list of parameters used in the proposed CNN architecture is given in Table 1.

The use of deep learning for image processing is recommended due to its generalization ability and the power to extract high-level features without any prior knowledge information or pre-processing steps. For instance, SAE (Stacked Auto Encoder) is found to be a good candidate for space reduction because of the close relationship between PCA properties such as orthogonality space, sensitivity to intra and inter variations of class, and meaningful representation of high dimensional data [39]. Recently, SAE was found to be a good candidate for space reduction because of its close relationship with PCA properties such as orthogonality space and sensitivity to intra and inter variations of class. The network topology consists of three layers: encoder layer, decoder layer, and output layer. All layers are fully connected through weight matrices, a bias vector, and a nonlinear function. Let us define the input vector $x\in R^{n}$ calculated at the hidden layer as in Equation (22).

(22) $$\begin{array}{c} h=f(W_{1}x+b_{h}) \end{array}$$

where f(x)=1/(1+exp(−x)) is the non-linear activation function applied component-wise, $F\left(x\right)\in R^{n}$ is the vector of node activation, $W_{1}\in mxn$ is a weight matrix, and $b\in R^{n}$ is a bias vector. The output layer y is given in Equation (23).

(23) $$\begin{array}{c} y=f(W^{T}h+b_{y}) \end{array}$$

where *y* is the reconstructed output. The cost function is computed as in Equations (24)–(26).

(24) $$\begin{array}{c} L\left(x,y\right)={∥x-y∥}^{2} \end{array}$$

(25) $$\begin{array}{c} J_{AE}\left(\theta \right)=\sum_{x\in D}L\left(x,g\left(f\left(x\right)\right)\right) \end{array}$$

(26) $$\begin{array}{c} J_{AE}\left(\theta \right)=\left[\frac{1}{m}\sum_{m}\left(\frac{1}{2}{∥x-y∥}^{2}\right)\right]+\frac{\lambda }{2}\sum_{l=1}^{2}\sum_{i}\sum_{j}W_{ij}^{\left(l\right)} \end{array}$$

where *D* is the set of training examples. Bias parameters are initialized to zero and weights are initialized as random uniform distribution [39]. However, achieving a high discriminality in the lower dimension based on minimizing reconstruction error is not enough. The authors incorporate a new constraint term into the cost function, called sparsity constraint. Thus, it leads to enforce the output of hidden layer activation to be equal to zero. As a result, the data in the new feature space can be given as in Equation (27).

(27) $$\begin{array}{c} {\hat{\rho }}_{j}=\frac{1}{\;m}\sum_{i=1}^{m}\left[a_{j}\left(x^{\left(i\right)}\right)\right] \end{array}$$

where ${\hat{\rho }}_{j}$ is the average activation of the j-th node of the i-th sample, m is the number of training set, and $\frac{1}{\;m}\sum_{i=1}^{m}\left[a_{j}\left(x^{\left(i\right)}\right)\right]$ denotes the j-th hidden node activation of the i-th sample. It is important to note that the average activation needs to be set as zero or close to zero. A penalty term is also added to the cost function. To deal with this purpose, the Kullback–Leibler (KL) divergence is a suitable tool to find the optimal parameters of the cost function under those constraints [39]. The KL divergence calculates the difference between two Bernoulli probability distributions of sparsity penalty (desired ${\hat{\rho }}_{j}$ and real $\rho_{j}$).

(28) $$\begin{array}{c} \sum_{j=1}^{s_{2}}\mathrm{KL}\left(\rho ∥{\hat{\rho }}_{j}\right)=\sum_{j=1}^{s_{2}}\rho \log\frac{\rho }{{\hat{\rho }}_{j}}+\left(1-\rho \right)\log\frac{1-\rho }{1-{\hat{\rho }}_{j}} \end{array}$$

By using the KL divergence, the new SAE cost function can be rewritten as in Equation (29).

(29) $$\begin{array}{c} J_{SAE}\left(\theta \right)=J\left(W,b\right)+\beta \sum_{j=1}^{S_{2}}KL\left(\rho ∥{\hat{\rho }}_{j}\right)=\frac{1}{\;m}\sum_{i=1}^{m}J\left(w,bx^{\left(i\right)},y^{\left(i\right)}\right)+\frac{\lambda }{2}\sum_{l=1}^{m_{1-1}}\sum_{i=1}^{S_{1}}\sum_{j=1}^{S_{t+1}}{\left(\;W_{j,i}^{\left(l\right)}\right)}^{2} \end{array}$$

To reconstruct the new output from the decoder within its parameters $\left\{W,b_{h},b_{y}\right\}$, the gradient descent algorithm (backpropagation learning and L2 regularization) is applied. In this study, the methodology for SAE-fused image model can be divided into five steps:

- Coding step: Once the SAE parameters such as W and b were initialized, number epochs, the iteration number, learning rate and sparsity parameter were well-tuned (i.e., the L2 regularization or the weight decay is well-tuned to force the hidden layers to be relatively small). By doing this, the first layer learns the fused image through a nonlinear activation layer and then trains it with unsupervised training.
- Decoding step: The second layer is trained by the corresponding SAE parameter values in order to reconstruct the first output layer while minimizing the reconstruction error.
- Stacked AE: Here, our network is made by stacking the SoftMax layer within the two previous layers.
- Fine-tuning step: After having pre-trained each layer’s AE separately, the back-propagation algorithm is used to optimize the whole stacked network parameters.
- Classification phase: The SoftMax with cross-entropy is expected to distinguish between the label classes either with or without anomaly.

The initial parameters of the proposed approach are represented in Table 2.

Deep Belief Networks (DBN) also obtain good results in image data classification. The DBN structure (i.e., probabilistic graph) and training procedure (unsupervised and greedy algorithm) makes it a successful DL classifier.

**Figure 4:**
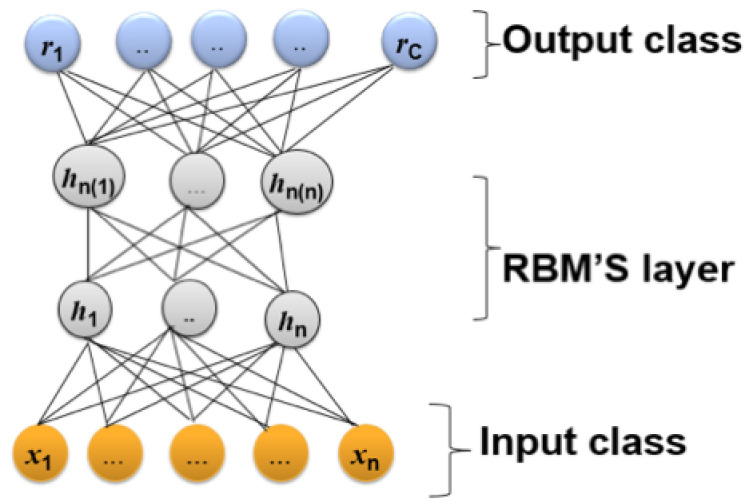
Diagram of a Deep Belief Network (DBN) architecture.

The operation of DBN can be understood by considering the theory of Boltzmann machines, particularly Restricted Boltzmann Machines (RBM). The Boltzmann machine is a bipartite graph in which their units (visible and hidden) are connected with unidirected weighted connections, as shown in Figure 4. Unlike the Boltzmann machine, there is no connection between the same units (i.e., visible–visible and hidden–hidden) [40]. In addition, the units are binary or stochastic. In the case of real data values, the hidden units are binary, and the visible units are computed as Gaussian distributions. RBMs train the stacked layer by applying unsupervised learning for the bottom layer and supervised learning for the top layer. RBMs identify the connection layer values as probability distribution values over the joint states of the visible and hidden units via an Boltzmann function as defined in Equation (30).

(30) $$\begin{array}{c} P\left(x,h_{1},h_{2},\ldots h_{n}\right)=\left(\prod_{k=0}^{n-2}P\left(h_{k}/h_{k+1}\right)\right)P\left(h_{n-1},h_{n}\right) \end{array}$$

where $x=h_{0}$, $P(h_{k}/h_{k+1})$ is a conditional distribution for the visible units conditioned upon the hidden units of the RBM at layer *k*, and $P\left(h_{n-1},h_{n}\right)$ is the visible–hidden joint distribution in the top-layer RBM. Figure 4 illustrates the structure of this model.

The DBN used in this work is described in Table 3.

The ELM method consists of a Single-Layer Feed-Forward Neural Network (SLFNN), which has a good generalization performance to improve the training step speed [41]. The aim of ELM is to find the unique solution among best solutions. Unlike a traditional feed-forward network, ELM’s training function guarantees the performance generalization and training speed. The ELM function can be summarized by Equations (31), (34)–(36).

Let us have randomly selected training samples of N elements, where $X_{i}=\left[x_{i1},x_{i2},\ldots \ldots x_{in}\right]\in R^{n}$ and $Y_{i}=\left[y_{i1},y_{i2},\ldots y_{im}\right]\in R^{m}$.

Where the activation function of hidden layer is g(X) and *L* is the hidden layer.

The first layer of ELM transfers the input data as in Equation (31).

(31) $$\begin{array}{c} \sum_{i=1}^{L}\beta_{i}g\left(X_{j}\right)=\sum_{i=1}^{L}\beta_{i}g\left(w_{i}\cdot X_{j}+b_{i}\right) \end{array}$$

The connection between layers (input to hidden layers) and (hidden to output layer) is defined by the weight vector (Equation (32)).

(32) $$\begin{array}{c} W_{i}=\left[W_{i1},W_{i2},\ldots \ldots W_{in}\right]\in Y_{i}\left(j=1,2,\ldots \ldots ,N\right) \end{array}$$

The weight vector connecting the input and hidden layer is randomly assigned. The bias vector is defined in Equation (33), where $B_{i}$ is the bias of the *i*–th hidden neuron (inner product of $W_{i}$ and $X_{j}$), the transfer function is g(x), $Y_{i}$ is the corresponding target vector, and g(x) the active function.

(33) $$\begin{array}{c} B_{i}=\left[B_{i1},B_{i2},\ldots \ldots B_{in}\right]\in Y_{i}\left(j=1,2,\ldots \ldots ,N\right)\;with\;Y_{i}=\left[Y_{i1},Y_{i2},\ldots \ldots Y_{in}\right] \end{array}$$

The last layer computes the inner product between the weight vector and the output of the previous layer. Here, the weight vector that connects the hidden and output layer is adjusted by finding the least-square solution of Equation (34).

(34) $$\begin{array}{c} H\beta =Y \end{array}$$

where H is defined in Equation (35).

(35) $$\begin{array}{c} H=\left[\begin{array}{ccc} g\left(w_{i}\times X_{j}+b_{i}\right) & \cdots & g\left(w_{i}\times X_{j}+b_{i}\right)\\ \vdots & \ddots & \vdots \\ g\left(w_{i}\times X_{j}+b_{i}\right) & \cdots & g\left(w_{i}\times X_{j}+b_{i}\right) \end{array}\right] \end{array}$$

Then, the weight matrix is computed in Equation (36), where $H^{+}$ is the Moore Penrose inversion of *H*.

(36) $$\begin{array}{c} \hat{\beta }=H^{+}Y \end{array}$$

In this case, the parameters used in the ELM algorithm were the following: (1) number of hidden neurons, H (h = 300, 500, 700, 900, 1000), and (2) neuron activation function, G (“Sigmoid”, “Triangular Basis”, “Sine”, “Hard Limit”, or “Radial Basis”) [41].

## 3. Results

Once the HSI image had been transformed into a 2D single image through fusion techniques, we applied deep learning techniques to train the transformed HSI images (with and without anomaly). The training followed a five-fold cross-validation procedure.

The performance of classifiers is assessed by accuracy (AC), precision (PR), recall (R), Cohens kappa coefficient (K), and F-measure (F), defined by Equations (37)–(40) [42], where $T_{p}$ = True positive, $T_{n}$ = True negative, $F_{p}$ = False positive, *P* = total number of positive samples, and *T* = total number of samples. A positive sample ($T_{p}$) is considered as a normal tray, and a negative $T_{n}$ is considered to have an anomaly.

(37) $$\begin{array}{c} accuracy=\frac{T_{p}+T_{n}}{T} \end{array}$$

(38) $$\begin{array}{c} recall=\frac{T_{p}}{P}\;\;\;;\;\;\;precision=\frac{T_{p}}{T_{n}+F_{p}} \end{array}$$

(39) $$\begin{array}{c} F_{measure}=\frac{2\times (precision\times recall)}{\left(precision\times recall\right)} \end{array}$$

(40) $$\begin{array}{c} K_{kappa}=\frac{2\times \left(T_{p}+T_{n}-F_{n}\times F_{p}\right)}{\left(T_{p}+F_{p}\right)\times \left(T_{n}+F_{p}\right)+\left(T_{p}+F_{n}\right)\times \left(T_{n}+F_{n}\right)} \end{array}$$

Results are also evaluated by the confusion matrix, which is a practical visualization for the outcome of model classification. It assigns each test sample to a predicted label based on the ground truth values. For example, in the case of average fusion, 13 food tray samples belonging to “without anomaly” category were assigned by the CNN classifier to the “anomaly class”.

**Figure 5:**
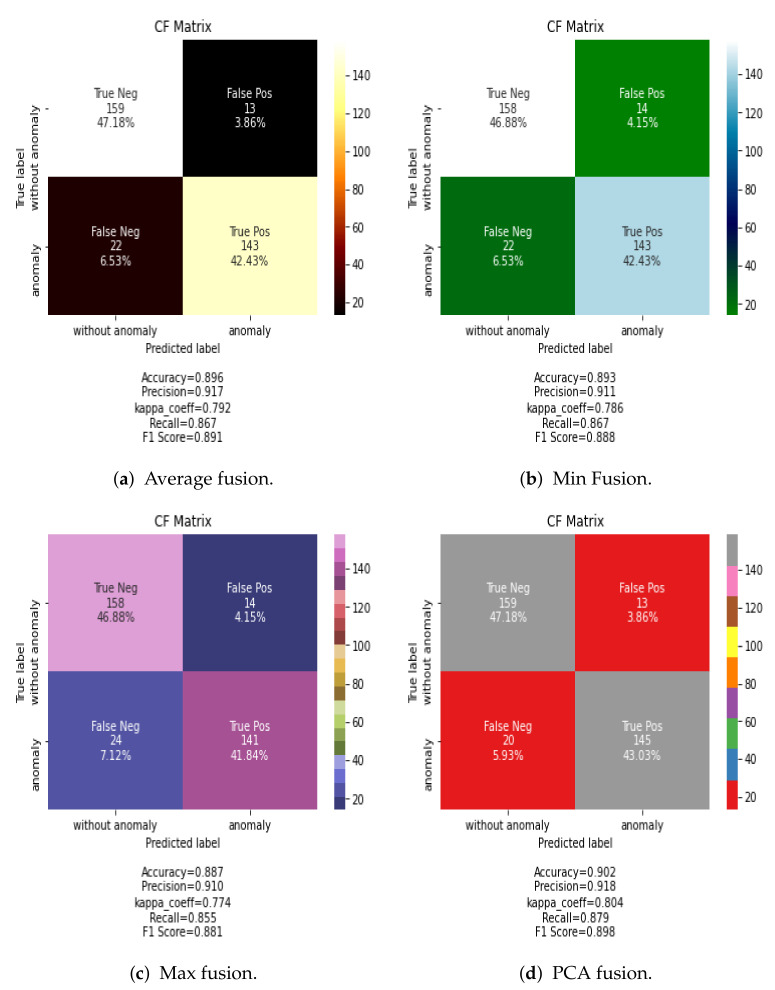
Confusion matrix for different spatial data fusion methods combined with the CNN classifier.

Figure 5 shows the confusion matrix for the classification results using the CNN classification algorithm and the proposed methods for data fusion in the spatial dimension. The PCA fusion method provides slightly better results than other spatial methods, showing that a selection of principal components as input data to the classifier provides better results than less advanced methods as maximum, minimum, or average.

**Figure 6:**
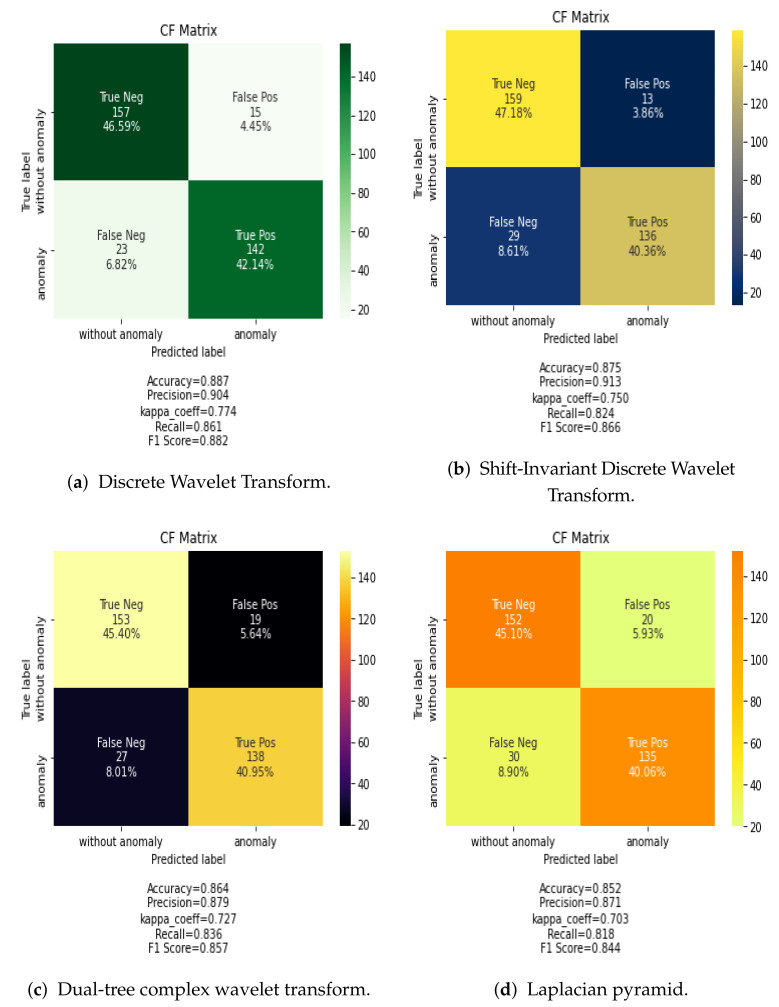
Confusion Matrix of reults for frequency data fusion methods combined with the CNN classifier.

Figure 6 shows the confusion matrix for the classification results using the CNN classification algorithm and the proposed methods for data fusion in the frequency dimension. In this case, DiscreteWavelet Transform and Shift-Invariant Discrete Wavelet Transform data fusion methods provide the best results. However, these results are lower than those of spatial data fusion methods.

**Figure 7:**
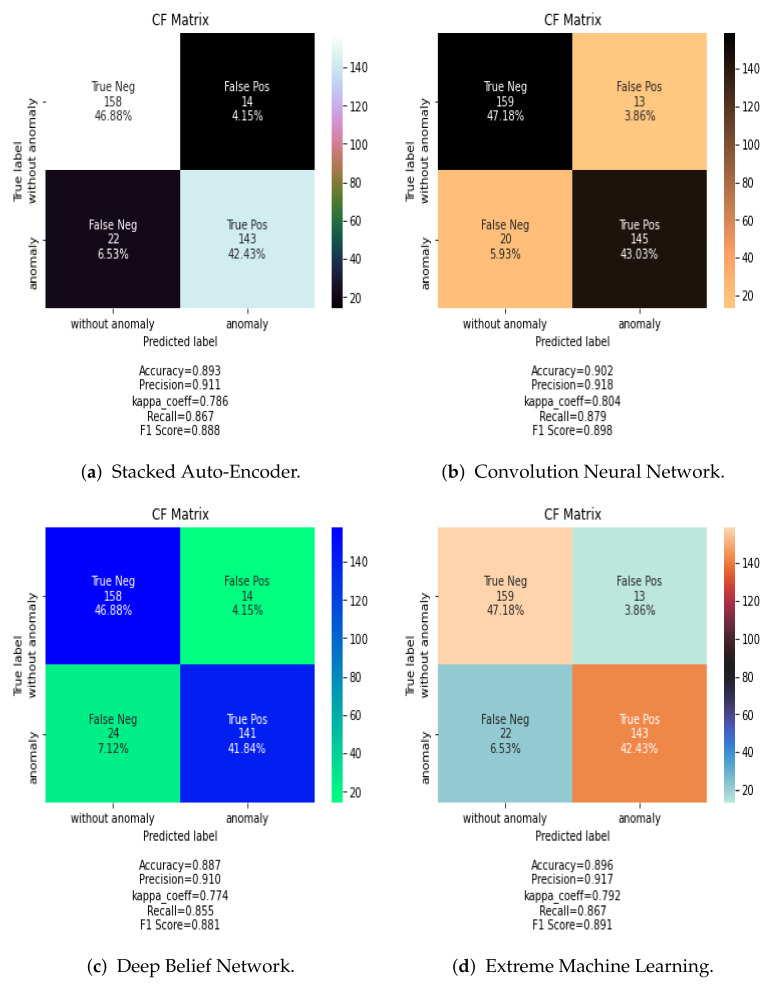
Confusion Matrix of SAE, CNN, DBN, and ELM classification methods using the PCA fusion rule.

In general, the CNN classifier obtains the best results among the analyzed DL classifiers. Figure 7 shows the confusion matrix results for SAE, CNN, DBN, and ELM classification algorithms using the PCA data fusion technique. In case of other data fusion techniques, the CNN always showed the best performance.

The results visualized in the confusion matrix in terms of accuracy (AC), precision (PR), recall (R), Cohens Kappa (K), and F-measure score (F) are summarized in Table 4 for spatial data fusion and Table 5 for frequency data fusion methods.

Evaluating the results shows that the CNN network combined with PCA data fusion provided the best accuracy. Generally, the PCA fusion rule can achieve a high detection rate for any classification method adopted in this study.

Table 4 shows that the CNN network combined with PCA data fusion provided the best accuracy (90%) in spatial data fusion, and Table 5 shows that the CNN combined with Discrete Wavelet Transform fusion rule obtains 86% accuracy, which outperforms other frequency data fusion techniques analyzed. This is due to the decomposition level into low frequency information within high-frequency information as well as the lower coefficient energies extracted from multi band modeling in the DWT. Overall, spatial data fusion provides better results than frequency data fusion. The full classification maps given in Figure 6 also demonstrate the effectiveness of Discrete Wavelet Transform combined with CNN network.

For example, in Table 4, PCA-CNN offers higher accuracy than other combinations. Moreover, spatial-fused methods (i.e., Min, Max, Average, PCA) achieve much higher classification accuracies than frequency fused-based methods (i.e., Dual Tree-CWT, Shift-Invariant DWT and Laplacian pyramid) except DWT. This clearly demonstrates that spatial fusion based methods keep the relevant features computed over the whole frequency bands.

As for the classification algorithms, the CNN classifier is found to be more effective either for spectral fusion or spatial fusion based methods. For example, in Table 4 and Table 5, the CNN has an overall classification accuracy above 85% while the classification accuracy of ELM, SAE and DBN are lower except for the Max fusion procedure.

In Table 6, the computational cost of CNN training step is higher than that of other classifiers due to the fact that it yields more layers and the number of parameters involved in each step. However, the computation is faster during the tests. It is worth mentioning that the computational complexity of spatial fusion methods is lower than that of spectral methods.

The ELM algorithm performs better when the selected fusion is frequency, and DBN performs better when the fusion is spatial.

## 4. Discussion

It is important to note that the data fusion method, together with the classifier, provides differences in the final classification accuracy. As already described in the literature, wavelength information is important for a successful classification. For this reason, data fusion in the spatial dimension show better results since no information is eliminated from the frequency domain.

As the HSI datacube requires heavy computation, we propose specific algorithms to keep the important information that leads us to obtain a classifier separating between food trays with seal anomaly and without anomalies. For this goal, the spatial and spectral information is essential, and the proposed data fusion procedures aim to decrease the of amount of information in pixels located in different bands, merging them into a single component that describes the pixel relationship in the ROI region.

Spectral fusion rules also represent different channel characteristics of some image as one single image, discarding the redundant information. The Discrete Wavelet and Laplacian pyramid methods are able to extract the relevant characteristics of multiple frequency bands. However, these methods suffer from higher computation cost when compared to spatial fusion.

## 5. Conclusions

To conclude, this paper shows a novel design for a food tray detection that benefits from the analysis of hyperspectral images but offers a high performance by using data fusion algorithms combined with deep learning algorithms in terms of accuracy and speed. Two kinds of fusion methods have been proposed to assess the gain in computational performance and accuracy. Additionally, the proposed deep learning algorithms do not require prior handcrafted features.

Generally, the performance of deep learning methods relies on the number of data used. Nowadays, it is not common to find HSI image database for the current purpose (food seal quality detection), and we could not find other databases to extend the application and evaluate the performance.

Future research work shall be to develop classification algorithms dealing with the full HSI datacube, which guarantees that no information is hidden by data fusion techniques, leading to improving the accuracy of the deep learning algorithms. However, a compromise between accuracy and computation time must be carefully considered, and only a significant increase in accuracy would justify the use of the whole HSI datacube, since we must consider that the food industry deals with a high tray processing speed (hundreds per minute), which means that the computation algorithm must be able to analyze the food tray while running in the production line.

## Figures and Tables

**Table 1:** Parameters of the CNN architecture used in this work.

Layer Type	Activation Unit	Kernel Size	Output Size
C1	ReLU	3×3	40×40×1
C2	ReLU	3×3	40×40×1
C3	ReLU	3×3	40×40×1
C4	ReLU	3×3	40×40×1
C5	ReLU	3×3	40×40×1
Flatten			
Fully-Connected			
Output	SoftMax		

**Table 2:** Different Stacked Auto Encoder (SAE) architectures used in this work.

Method	SAE
Number of Hidden layers	C1 C2 C3
Maximum number of epochs	100∼800
L2 Weight Regularization	0.001∼0.006
Sparsity Regularization	1∼4
Sparsity Proportion	0.1∼0.8

**Table 3:** Parameters used for the Contrastive Divergence (CD) DBN architecture

Method	Contrastive Divergence (CD)
Number of Hidden layers	C1 C2 C3 C4 C5
Maximum number of epochs	100∼800
Number of mini batches	1∼10
Batch size	1∼5
Momentum	0∼8
Learning rate	0.01∼0.06

**Table 4:** Accuracy (AC), Precision (PR), Recall (R), Cohens kappa (K), and F-measure (F) values in percentage for the analyzed spatial fusion methods and classification algorithms using spatial fused data as input.

	DBN					CNN				
	AC	PR	R	K	F	AC	PR	R	K	F
Average	88.7	90.0	77.4	86.1	88.2	89.6	90.7	79.2	86.7	89.1
Min	88.7	90.0	77.4	86.1	88.2	89.3	91.1	78.6	86.7	88.8
Max	88.3	90.0	77.2	86.0	88.1	88.7	90.0	77.4	86.1	88.2
PCA	88.7	91.0	77.4	85.5	88.1	90.0	91.8	80.0	87.9	89.8
	SAE					ELM				
	AC	PR	R	K	F	AC	PR	R	K	F
Average	87.3	91.0	74.9	82.1	86.4	88.3	90.0	77.2	86.0	88.1
Min	88.0	90.0	76.8	72.4	80.9	87.3	91.0	74.9	82.1	86.4
Max	88.3	90.0	77.2	86.0	88.1	88.3	90.0	77.2	86.0	88.1
PCA	89.3	91.1	78.6	86.7	88.8	89.6	91.7	79.2	86.7	89.1

**Table 5:** Accuracy (AC), Precision (PR), Recall (R), Cohens kappa (K), and F-measure (F) values in percentage for the analyzed spatial fusion methods and classification algorithms using frequency fused data as input.

	DBN					CNN				
	AC	PR	R	K	F	AC	PR	R	K	F
DWT	86.4	87.9	72.7	83.6	85.7	88.7	90.0	77.4	86.1	88.2
Dual-tree CWT	84.4	85.8	68.8	79.8	82.8	86.4	87.9	72.7	83.6	85.7
Shift-Invariant DWT	83.3	85.0	68.6	79.6	81.5	87.5	91.3	75.0	82.4	86.6
Laplacian pyramid	83.4	85.0	68.8	79.8	81.8	85.2	87.1	70.0	81.8	84.4
	SAE					ELM				
	AC	PR	R	K	F	AC	PR	R	K	F
DWT	85.2	87.1	70.0	81.8	84.4	86.4	87.9	72.7	83.6	85.7
Dual-tree CWT	83.5	85.0	68.9	79.8	82.2	87.5	91.3	75.0	82.4	86.6
Shift-Invariant DWT	84.4	85.8	68.8	79.8	82.8	85.2	87.1	70.0	81.8	84.4
Laplacian pyramid	83.5	85.0	68.9	79.8	82.2	84.4	85.8	68.8	79.8	82.8

**Table 6:** Computation time for the analyzed classifiers. Total training time and average computation time for one test sample (HSI datacube).

Computation Time	Total Train Time (s)	Avg. Test Time (s)
DBN	220.5	0.33
CNN	500.5	0.90
SAE	250.8	0.47
ELM	300.0	0.60

## Data Availability

Not applicable.
